# Acute immunometabolic changes in first-presentation Graves’ hyperthyroidism patients undergoing strenuous physical activity

**DOI:** 10.3389/fendo.2025.1685496

**Published:** 2025-11-21

**Authors:** Yuqian Ren, Zhenchao Liu, Meng Wang, Yanzhi Wang, Yun Wang

**Affiliations:** 1 School of Basic Medicine, Qingdao University, Qingdao, Shandong, China; 2 School of Pharmacy, Qingdao University, Qingdao, Shandong, China; 3 Department of Pharmacy, Linyi People’s Hospital, Linyi, Shandong, China; 4 Office of Academic Affairs, Binzhou Medical University, Yantai, Shandong, China; 5 Health Management Center, Linyi People’s Hospital, Linyi, Shandong, China

**Keywords:** Graves’ hyperthyroidism, high-intensity intermittent exercise, immunometabolic response, muscle mass, thyroid hormones

## Abstract

**Objective:**

This study systematically evaluated immunometabolic responses in newly diagnosed female Graves’ hyperthyroidism patients versus healthy controls following repeated high-intensity intermittent exercise (HIIE, Wingate test). It also preliminarily explored the associations of thyroid hormone levels and muscle mass with these responses.

**Methods:**

We enrolled 25 newly diagnosed female Graves’ hyperthyroidism patients (observation group) and 25 healthy women (control group). All participants underwent three HIIE. Blood samples, collected before and immediately after the third test, were analyzed for glucose, lactate, leptin, irisin, creatine kinase (CK), interleukin-6 (IL-6), interleukin-15 (IL-15), and tumor necrosis factor-alpha (TNF-α) levels. Baseline skeletal muscle index (SMI) and thyroid hormone levels were also measured. Data were analyzed using repeated measures ANOVA, Bayesian repeated measures ANOVA, and Bayesian network analysis.

**Results:**

Baseline data indicated that the observation group exhibited significant higher free triiodothyronine (FT3) and free thyroxine (FT4), and lower thyroid-stimulating hormone (TSH) (*P* < 0.001). Additionally, this group demonstrated a markedly reduced SMI. (*P* = 0.005). Both peak and mean HIIE power significant decreased with increasing repetitions in both groups (*P* < 0.001). While peak power decline showed no significant group difference, mean power decline revealed a significant “Times x Group interaction” (*P* < 0.001), indicating distinct fatigue patterns. Metabolically, lactate and glucose significant increased post-HIIE in both groups (*P* < 0.001), with no significant group differences in their trends. Leptin significant increase in both groups (*P* < 0.001), and Bayesian analysis indicated significant inter-group differences. CK and irisin significant increased post-HIIE (*P* < 0.001) with no significant group differences in levels or trends. Inflammatory markers showed that IL-6 had a significant average level difference between groups (*P* < 0.001) and increased significant post-HIIE (*P* < 0.001), with a significant interaction in its increase trend (*P* = 0.017). IL-15 and TNF-α exhibited significant average level differences (*P* < 0.05) but no significant post-HIIE changes or group differences in trends. Bayesian network analysis in the hyperthyroidism group revealed complex interrelationships among FT3, FT4, TSH, IL-15, leptin, IL-6, and TNF-α, with TSH significant regulating inflammatory responses and SMI potentially influencing pre- and post-exercise glucose levels. The control group network was simpler.

**Conclusion:**

This study, for the first time, systematically evaluated immunometabolic responses in female Graves’ hyperthyroidism patients following HIIE. While muscle loss in newly diagnosed hyperthyroidism patients did not significant impair short-term explosive power, it may reduce endurance during sustained high-intensity exercise. The hyperthyroid state markedly altered exercise-induced immunometabolic dynamics, underscoring the need for comprehensive consideration of hyperthyroidism’s complex physiological impact when designing personalized exercise regimens.

## Graphical Abstract

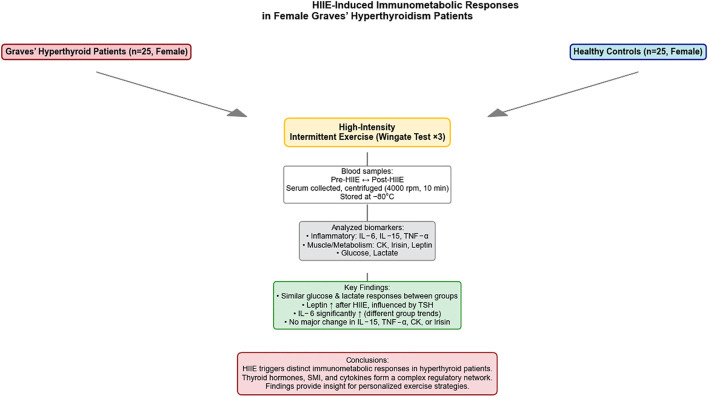

## 1 Introduction

High-intensity intermittent exercise (HIIE) profoundly impacts human physiological systems in multiple dimensions. Its performance is comprehensively regulated by complex factors, including the effective supply of energy substrates, maintenance of acid-base balance, key enzyme activity, and the synergistic action of different energy systems (1). During acute exercise stress, changes in the levels of endocrine and immune mediators such as creatine kinase (CK) and interleukin-6 (IL-6) can regulate energy substrate availability. These changes are further influenced by factors such as exercise type, intensity, and duration (1–3). Exercise-induced immunometabolic responses are closely linked to glucose homeostasis in muscle cells (4). Notably, the acute increase in exercise-induced IL-6 is considered beneficial for human health due to its potential anti-inflammatory effects (5).

Previous research on exercise-induced cytokine responses has primarily focused on alterations in inflammatory cytokines, particularly IL-6 and tumor necrosis factor-alpha (TNF-α), following long-term exercise and resistance training in healthy individuals (6, 7). Pro-inflammatory cytokines, for instance, can promote various metabolic events, including lipolysis and glycogenolysis, during acute or chronic exercise training, thereby facilitating the adequate supply of substrates for exercise. Conversely, anti-inflammatory cytokines exert a protective effect by counteracting persistent inflammatory responses (8–10). However, there is a relatively limited number of studies specifically investigating the impact of acute exercise on metabolic and inflammatory profiles in individuals with metabolic abnormalities.

Hyperthyroidism is a common endocrine disorder characterized by excessive thyroid hormone secretion, leading to an abnormally elevated metabolic rate, often accompanied by symptoms such as sarcopenia, increased heart rate, and elevated energy expenditure (11). These physiological changes may directly affect the ability of hyperthyroid patients to engage in physical activity and their response to exercise stress. Previous research indicates that the hyperthyroid state, particularly muscle loss, can lead to decreased exercise tolerance, increased susceptibility to fatigue, and potentially place an additional burden on the cardiovascular system (12, 13). However, a more comprehensive and systematic in-depth analysis is still needed regarding the dynamic changes in immunometabolic responses in hyperthyroid patients after continuous high-intensity exercise, as well as the correlation of these changes with potential physiological indicators such as thyroid hormone levels and body composition (especially muscle mass). To date, no study has systematically evaluated the effect of continuous high-intensity intermittent exercise on the immunometabolic profile in hyperthyroid patients and compared it with healthy controls. Therefore, this study aims to assess the similarities and differences in immunometabolic factors between hyperthyroid patients and healthy controls after continuous HIIE (Wingate test). We hypothesize that although HIIE in both populations may lead to increased energy substrate availability and cytokine levels, the physiological response pattern in hyperthyroid patients will significant differ from that of healthy controls, particularly concerning metabolic products and muscle-related cytokines, due to their unique metabolic state and muscle loss.

In selecting the exercise assessment methodology at the outset, our rationale was based on the characteristic alterations observed in skeletal muscle fibers under hyperthyroid conditions: the protein expression of slow-twitch fibers (MHC-I) is markedly reduced and shows minimal improvement even with exercise stimulation, whereas the fiber composition shifts toward fast-twitch fibers (MHC-2a) with a limited capacity for fast-to-slow fiber conversion. This phenomenon is believed to be associated with the long-term effect of thyroid hormones in promoting the transition from MHC-I to MHC-2a, as well as the persistently elevated baseline activation of the mTOR signaling pathway, which restricts the potential for additional exercise to further enhance protein synthesis or fiber remodeling (14, 15). Given that fast-twitch fibers predominantly rely on anaerobic energy supply during short-duration (16), high-intensity exercise, assessment of anaerobic metabolic capacity can more accurately reflect the muscle functional characteristics and adaptability of hyperthyroid patients. On this basis, the present study adopted the Wingate test—a widely used method for evaluating anaerobic metabolism—which enables simultaneous measurement of peak power (indicative of phosphagen system capacity) and mean power (reflecting the combined contributions of the phosphagen and glycolytic systems) (17), thereby providing a comprehensive characterization of muscle energy supply features under short-term, high-intensity load in hyperthyroid individuals. In this study, we re-analyzed the original first-phase data using Bayesian statistical methods, revealing entirely new and clinically relevant findings that had not been detected through conventional frequentist approaches, thereby offering fresh insights into the muscle metabolism of patients with hyperthyroidism.

## 2 Methods

### 2.1 Study participants

**Figure 1:**
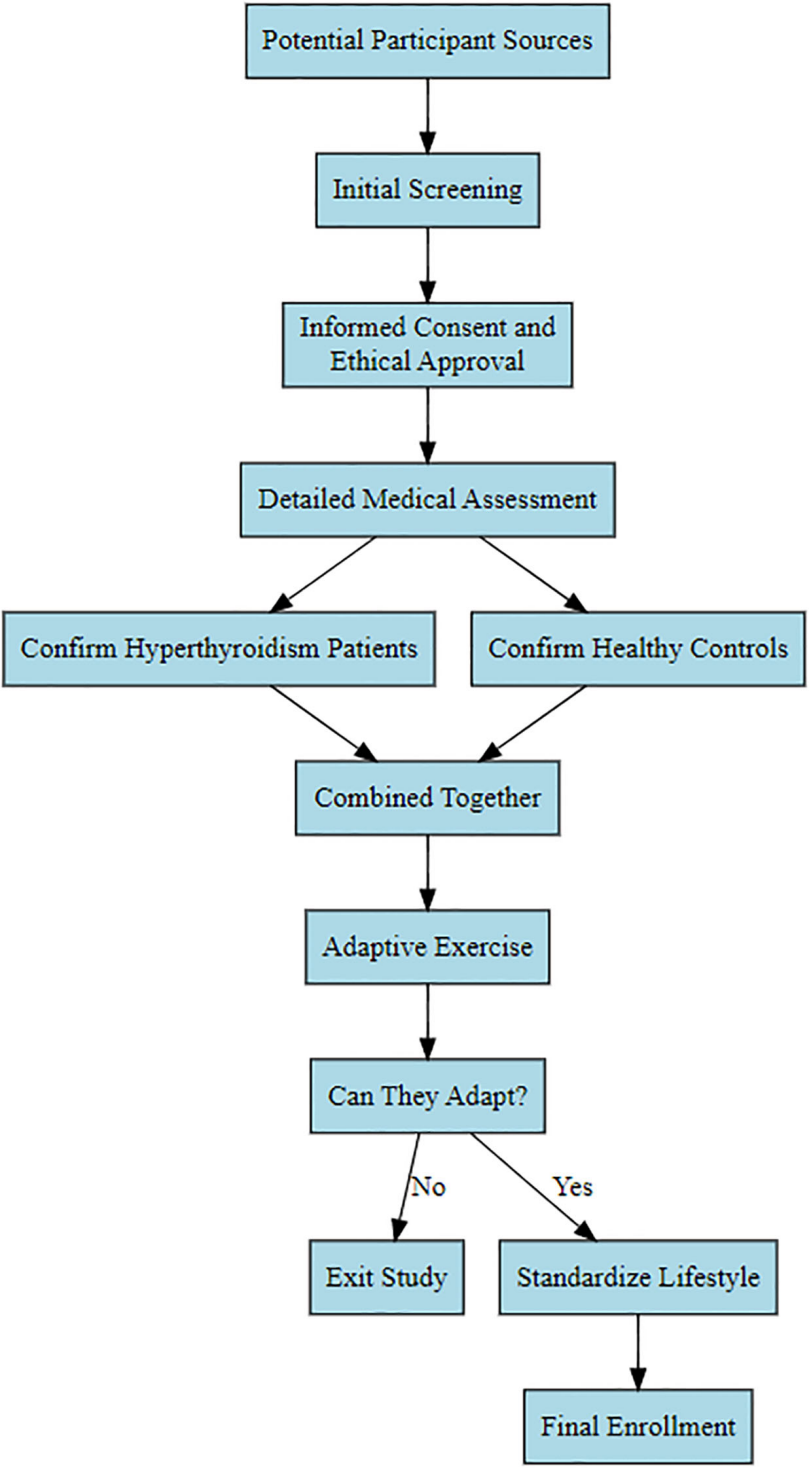
Participant enrollment flowchart.

First-visit Graves’ hyperthyroidism patients admitted to the East Medical District of Linyi People’s Hospital in 2017 were selected as the observation group. All participants were enrolled after screening based on inclusion and exclusion criteria. Following enrollment, participants underwent adaptive exercise for the Wingate test; those unable to participate were withdrawn. Ultimately, 25 female participants were selected for the study. Due to the relatively small number of male patients and fewer willing male participants, the study exclusively focused on female Graves’ hyperthyroidism patients. Subsequently, 25 healthy females were selected as a control group based on inclusion/exclusion criteria and adaptive exercise (refer to **Figure 1** for the specific screening process). This study received approval from the Medical Ethics Committee of Linyi People’s Hospital (Ethics Approval No.: KY2017015). All participants voluntarily joined the study after reviewing the informed consent form. They all maintained a consistent lifestyle for one week, during which patients were prohibited from consuming high-sugar, high-fat diets or snacks. Daily sleep was from 23:00 to 7:00 the following day, and strenuous physical activity and exercise training were avoided.

#### 2.1.1 Inclusion criteria

1. Female participants aged 18 years or older;

2. Patients were newly diagnosed with Graves’ hyperthyroidism. Inclusion criteria for the observation group were: confirmed diagnosis of hyperthyroidism, with serum thyroid-stimulating hormone (TSH) below the lower reference limit, and serum free thyroxine (FT4) or total thyroxine (TT4) above the upper reference limit. Inclusion criteria for the healthy control group were: normal thyroid function, i.e., TSH, FT4, and TT4 levels all within the normal reference range, and no history of other endocrine system diseases or chronic diseases;

3. All participants were required to voluntarily sign informed consent, understand and be able to follow the research protocol, successfully complete the Wingate test adaptive exercise, and be able to participate in the formal experiment.

#### 2.1.2 Exclusion criteria

1. Individuals with cardiovascular, liver, kidney, diabetes, or other metabolic diseases, as well as autoimmune diseases (excluding thyroid autoimmune diseases) or any other known conditions that might affect exercise response or metabolic indicators;

2. Individuals with neuromuscular or osteoarticular diseases that might affect exercise capacity or increase the risk of exercise injury;

3. Individuals with recent acute infections or inflammatory responses;

4. Individuals currently taking medications that might influence metabolic or inflammatory responses (e.g., glucocorticoids, non-steroidal anti-inflammatory drugs), as well as those who smoke, abuse alcohol, or have a history of drug abuse;

5. Pregnant or lactating women;

6. Participants who were unable to complete the Wingate test adaptive exercise or the formal test, or who could not adhere to the unified lifestyle pattern during the study period (including dietary, sleep, and physical activity restrictions), would also be excluded;

7. For the hyperthyroid patient group, participants with unstable conditions, those undergoing treatment regimen adjustments, or those with severe hyperthyroid complications were excluded.

### 2.2 Methods

#### 2.2.1 Measurement of skeletal muscle mass

Whole-body fat-free mass was measured using a dual-energy X-ray absorptiometry (DXA) densitometer (Lunar Prodigy, GE, USA). The DXA operating procedure was initiated, and a whole-body dual-energy X-ray absorption scan was selected. After measurement, SMI = appendicular skeletal muscle mass/height (kg/m^2^) was calculated.

#### 2.2.2 HIIE test

Before the test, all participants avoided strenuous exercise for 24 hours. Then, three intermittent high-intensity Wingate tests (Monark, 818E, Sweden) were conducted with 5-minute intervals. First, participants warmed up for 15 minutes on an indoor road bicycle. After warming up, participants pedaled the bicycle as fast as possible twice until their heart rate exceeded 150 beats/min, rested for 5 minutes until their heart rate dropped to usual resting level, and then proceeded to the test using the 818E power ergometer. Upon the start signal, participants were instructed to pedal as fast as possible for 30 seconds, remaining seated throughout the test, and were encouraged to exert maximal effort. Peak power (the maximum power attained during the 30-second test) and average power (the average power generated during the 30-second test) were recorded. After a 5-minute rest, a second Wingate test was performed, followed by another 5-minute rest, and then a third Wingate test. The specified resistance was set at 75g/kg. Blood samples were collected from participants immediately before the test and immediately after the third test.

#### 2.2.3 Measurement of IL-6, IL-15, and TNF-α levels

Immediately before the test and immediately after the third high-intensity Wingate test, 2 mL of venous blood was drawn from the cubital vein of the upper arm using standard vacuum tubes without any anticoagulant additives. Samples were centrifuged at 4,000 rpm for 10 min at low speed to separate the serum. The supernatant was carefully transferred using a sterile separation pipette and stored at −80°C in an ultra-low temperature freezer, avoiding repeated freeze–thaw cycles. Serum levels of IL-6 (SUB10377), IL-15 (SUB11237), and TNF-α (SUB11776) were measured using commercially available enzyme-linked immunosorbent assay (ELISA) kits (BIOSH, China) according to the manufacturer’s instructions.

#### 2.2.4 Measurement of Leptin, Irisin, and CK levels

For each participant, 2 mL of venous blood was drawn from the cubital vein immediately before the test and immediately after the third Wingate test, following the same serum collection procedure described above. Serum concentrations of Leptin (EIA-0784H), Irisin (EIA-0016), and Creatine Kinase (CK, EIA-0935) were determined using ELISA kits from Elsbiotech (China).

#### 2.2.5 Measurement of blood glucose and blood lactate levels

Before the start of the experiment and 1 minute after each Wingate test, capillary blood samples were collected from the earlobe using a sterile lancet for lactate testing. Serum lactate concentration was immediately measured using an Accutrend Plus analyzer and recorded in mmol/L. Additionally, fingertip capillary blood samples from the middle finger were collected before and after the experiment using a lancet for glucose testing with an ACCU-CHEK glucometer, with results recorded in mmol/L.

### 2.3 Statistical analysis

This study utilized R 4.5.0 for data analysis. First, Given the research objective based on muscle level differences, an *a priori* power analysis was conducted using the difference in Skeletal Muscle Index (SMI) between two groups in our pilot data. Group 1 (n = 25) had a mean of 5.57 and a standard deviation of 0.68; Group 2 (n = 25) had a mean of 6.08 and a standard deviation of 0.58. Based on these data, the pooled standard deviation was calculated as 0.609, yielding a Cohen’s d of 0.841 (a large effect size). Setting α = 0.05 (two-sided) and desired power = 0.80, the minimum required sample size was estimated at 24 participants per group. The observed effect size for SMI between groups was Cohen’s d = 0.841. A *post hoc* power analysis with the actual sample size (n = 25 per group) yielded a statistical power of 0.829, exceeding the conventional threshold of 0.80. Therefore, the sample size was deemed adequate to detect the observed between-group difference. The Shapiro-Wilk test was used to assess the normality of the data, and related quantitative parameters were compared between groups using *t*-tests and rank sum tests. Then, repeated measures ANOVA was employed to evaluate the impact of relevant factors on changes in peak power, average power, blood glucose, blood lactate, leptin, irisin, CK, IL-6, IL-15, and TNF-α levels. Mauchly’s test was used to check for sphericity, and Geisser-Greenhouse correction was applied when necessary. If significant differences were found in the ANOVA, Bonferroni correction was used for *post-hoc* multiple comparisons. Furthermore, Bayesian repeated measures ANOVA was used to assess the effects of different groups and measurement conditions on each indicator. The model was set as a normal distribution, and prior distributions for relevant parameters were specified accordingly. Response variables were the various test indicators, with “group” and “times” as fixed effects, and variables representing inter-group differences included as covariates in the model. During model fitting, 4000 iterations and 4 MCMC chains were set to ensure the stability and convergence of the results, thereby evaluating the effects of multiple factors and their interactions. A Bayesian network was constructed using Bayesian methods, and the Hill-Climbing algorithm was applied to learn the Bayesian network structure from the data of both the observation and control groups. This algorithm was used to calculate and compare the network structure complexity of the two groups, including the number of nodes and edges. Additionally, we calculated the degree and betweenness centrality of each node to evaluate the key variables in the network.

## 3 Results

### 3.1 Comparison of baseline characteristics

Apart from differences in thyroid-related hormones (thyroid hormone FT3 and FT4 levels were significant higher in the observation group than in the control group, *P* < 0.001; while TSH levels were significant lower in the observation group than in the control group, *P* < 0.001), the observation group’s SMI was significant lower than that of the control group (*P* = 0.005). Other parameters such as age, BMI, blood glucose, cholesterol, low-density lipoprotein, and high-density lipoprotein showed no significant differences. (See **Table 1**).

**Table 1:** Comparison of baseline characteristics between two groups of participants.

Parameter	Control group	Observation group	*T/z*	*P*	95%CI
Age (years)	40.52±9.06	39.72±8.05	0.33	0.743	-4.08~2.42
BMI (kg/m^2^)	23.02±2.99	23.24±3.84	0.22	0.823	-2.18~0.97
SMI (kg/m^2^)	6.08±0.58	5.57±0.63	2.97	0.005	0.16~0.17
FT3 (pg/ml)	4.98±0.30	22.01±10.15	8.38	<0.001	-21.23~-12.84
FT4 (pg/ml)	15.93±0.89	58.07±30.31	6.95	<0.001	-54.33~-29.62
TSH (mIU/L)	1.95 (0.43)	0.01 (0.007)	6.07	<0.001	–
Blood Glucose (mmol/L)	5.27±0.61	5.33±0.59	0.35	0.727	-0.40~0.17
Total Cholesterol (mmol/L)	4.01±0.95	3.47±1.11	1.87	0.068	-0.04~1.13
Triglycerides (mmol/L)	1.22 (0.83)	1.03 (0.99)	0.18	0.854	–
LDL (mmol/L)	1.92±0.64	1.81±0.73	0.55	0.588	-0.29~0.20
HDL (mmol/L)	1.43±0.66	1.17±0.31	1.80	0.078	-0.03~0.15
Systolic BP (mmHg)	122.64±10.55	118.16±9.09	1.61	0.114	-1.12~10.08
Diastolic BP (mmHg)	79.12±9.94	76.76±6.18	1.01	0.318	-2.35~2.34

BMI, Body Mass Index; SMI, Skeletal Muscle Index; FT3, Free Triiodothyronine; FT4, Free Thyroxine; TSH, Thyroid Stimulating Hormone; LDL, Low-Density Lipoprotein; HDL, High-Density Lipoprotein; Systolic BP, Systolic Blood Pressure; Diastolic BP, Diastolic Blood Pressure.

### 3.2 HIIE power comparison

For peak power, the main effect of group was not significant (*F* = 1.599, *P* = 0.212, *η²* = 0.032), indicating no statistically significant difference in peak power levels between the two groups across the three tests. The main effect of “Times” was significant (*F* = 410.698, *P* < 0.001, *η²* = 0.895), revealing a significant difference across the three peak power measurements, meaning that participants’ peak power significant decreased as the number of tests increased. The interaction effect between “Times” and “Group” was not significant (*F* = 1.269, *P* = 0.278, *η²* = 0.026), indicating no statistically significant difference in the trend of peak power decline between the different groups across the three tests. Further within-subjects contrast analysis showed that the linear trend of “Times” was significant (*F* = 634.394, *P* < 0.001, *η²* = 0.930), suggesting a significant linear decrease in peak power with increasing test repetitions. The quadratic trend was also significant (*F* = 33.975, *P* < 0.001, *η²* = 0.414), indicating that the decline trend was not perfectly linear and exhibited some curvilinear changes. The linear and quadratic trends of the interaction between “Times” and “Group” were both not significant, further supporting the conclusion that there was no significant difference in the peak power decline pattern between the two groups. We included FT3, FT4, TSH, and SMI as interactive effects in the Bayesian RM-ANOVA. The results showed that after including these predictors, group (*β* = -0.62, 95% CI=-10.20~9.19), SMI (*β* = 5.02, 95% CI=-4.59~14.57), FT3 (*β* = -0.88, 95% CI=-4.76~3.09), FT4 (*β* = 0.77, 95% CI=-0.66~2.21), and TSH (*β* = -0.28, 95% CI=-9.74~9.05) had no statistically significant impact on peak power. (See **Table 2**).

**Table 2:** Peak power and average power of wingate test for two groups of participants.

Wingate test power (W)	Control group	Observation group
First Peak Power	444.68 ± 103.78	413.68 ± 133.64
Second Peak Power	396.00 ± 100.47	356.12 ± 122.93
Third Peak Power	299.64 ± 89.15	251.28 ± 123.60
First Average Power	362.72 ± 99.44	375.16 ± 96.66
Second Average Power	265.68 ± 81.61	294.96 ± 92.24
Third Average Power	206.76 ± 77.30	166.72 ± 95.45

The main effect of group on average power was not significant (*F* = 0.001, *P* = 0.982, *η²* < 0.001), indicating no statistically significant difference in average power levels between the two groups across the three tests. The main effect of “Times” was significant (*F* = 381.635, *P* < 0.001, *η²* = 0.888), showing a significant difference across the three average power measurements, meaning that participants’ average power significant decreased as the number of tests increased. The interaction effect between “Times” and “Group” was significant (*F* = 15.024, *P* < 0.001, *η²* = 0.238), indicating a significant difference in the trend of average power decline between the different groups across the three tests. Further within-subjects contrast analysis showed that the linear trend of “Times” was significant (*F* = 593.237, *P* < 0.001, *η²* = 0.925), suggesting a significant linear decrease in average power with increasing test repetitions. However, the quadratic trend was not significant (*F* = 0.264, *P* = 0.610, *η²* = 0.005), which differs from the peak power analysis results and suggests that the decline in average power was more linear. Both the linear trend (*F* = 12.304, *P* = 0.001, *η²* = 0.204) and the quadratic trend (*F* = 19.925, *P* < 0.001, *η²* = 0.293) of the “Times” by “Group” interaction were significant, indicating significant differences in both the linear slope and non-linear pattern of average power decline between the two groups. The Bayesian RM-ANOVA results showed that after including the predictors, all predictors—group (*β* = 0.52, 95% CI=-9.00~10.10), SMI (*β* = 4.99, 95% CI=-4.48~14.35), FT3 (*β* = -1.17, 95% CI=-4.44~2.06), FT4 (*β* = 0.34, 95% CI=-0.82~1.53), and TSH (*β* = 2.54, 95% CI=-6.74~11.87)—had no statistically significant impact on average power. (See **Table 2**).

### 3.3 Metabolic indicators comparison

**Figure 2:**
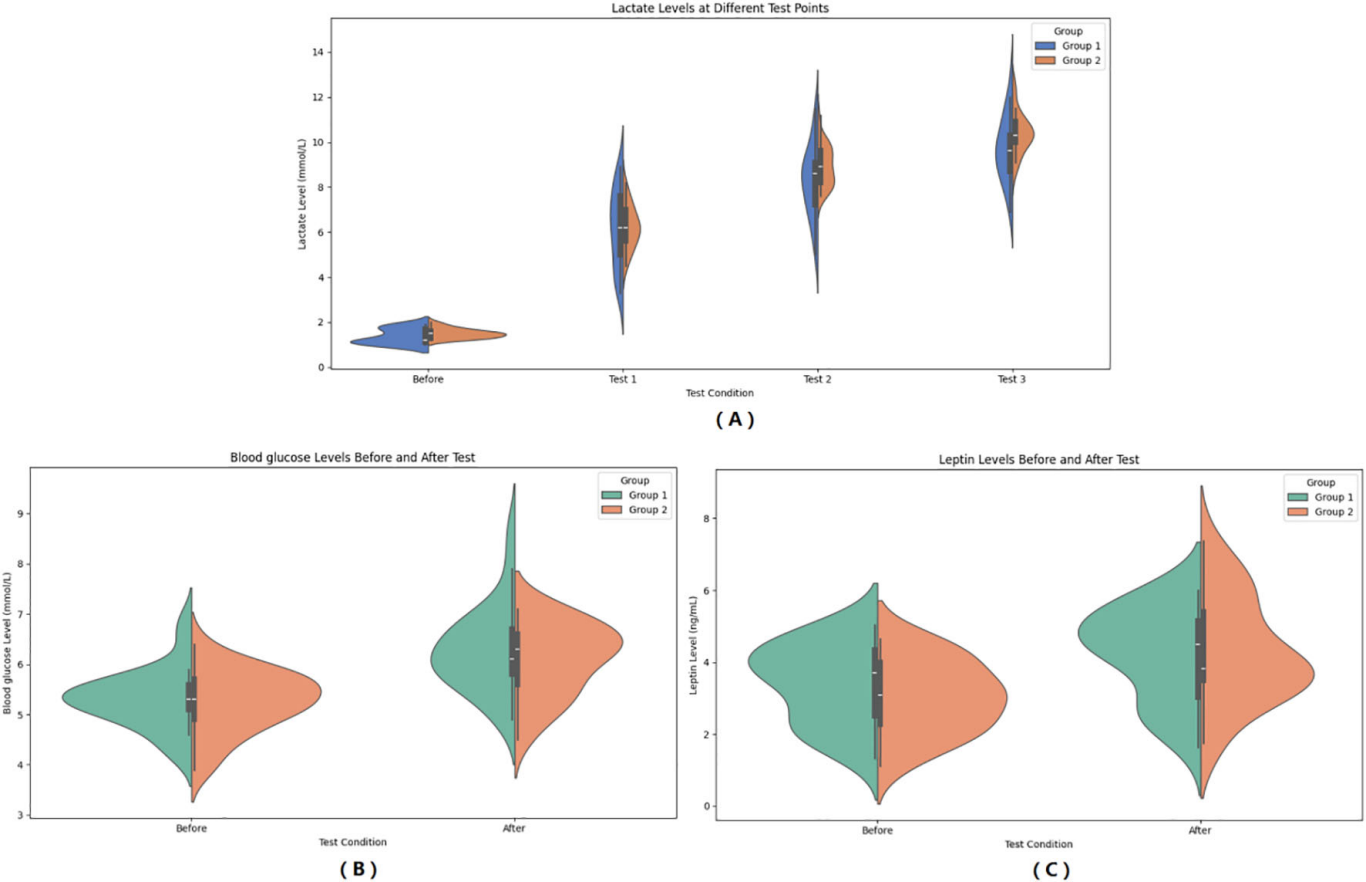
Pre- and Post-HIIE changes in metabolic indicators: Blood Lactate **(A)**, Blood Glucose **(B)**, and Leptin **(C)**. **(A)** Changes in blood lactate levels at different test points (Before, Test 1, Test 2, Test 3). **(B)** Comparison of blood glucose levels before and after HIIE. **(C)** Comparison of serum leptin levels before and after HIIE. Group 1: Control group; Group 2: Graves’ disease group. Data are presented as violin plots showing the distribution, median, and interquartile range.

The main effect of group on blood lactate was not significant (*F* = 2.602, *P* = 0.113, *η²* = 0.051), indicating no statistically significant difference in blood lactate levels between the two groups across the four tests. The main effect of “Times” was significant (*F* = 923.837, *P* < 0.001, *η²* = 0.951), which demonstrates a significant difference in the average lactate levels across the four tests, meaning that participants’ lactate levels significant increased as the number of tests increased. The interaction effect between “Times” and “Group” was not significant (*F* = 1.779, *P* = 0.171, *η²* = 0.036), indicating no statistically significant difference in the increasing trend of blood lactate levels between the different groups. Further within-subjects contrast analysis showed that the linear trend of “Times” was significant (*F* = 1885.126, *P* < 0.001, *η²* = 0.975), indicating a significant linear increase in blood lactate with increasing test repetitions. The quadratic trend was also significant (*F* = 155.754, *P* < 0.001, *η²* = 0.764), suggesting that the increasing trend was not entirely linear and exhibited some curvilinear changes. The cubic trend was also significant (*F* = 12.417, *P* = 0.001, *η²* = 0.206), further indicating that the lactate increase curve might be more complex. The linear, quadratic, and cubic trends of the “Times” by “Group” interaction were all not significant (*P*>0.05), further supporting the conclusion that there was no significant difference in the lactate increase pattern between the two groups. Bayesian RM-ANOVA results showed that after including the predictors, all predictors—group (*β* = -0.12, 95% CI=-1.781.56), SMI (*β* = 0.002, 95% CI=-0.280.28), FT3 (*β* = 0.01, 95% CI=-0.030.05), FT4 (*β* = 0.001, 95% CI=-0.010.01), and TSH (*β* = 0.34, 95% CI=-0.50~1.18)—had no statistically significant impact on lactate. (See **Table 3**; **Figure 2**).

**Table 3:** Changes in blood lactate levels in two patient groups before and after three wingate tests ( $\bar{\text{x}}$ ± s).

Blood lactate(mmol/l)	Control group	Observation group
Pre-HIIE	1.50 ± 0.21	1.38 ± 0.34
First Wingate Test	6.24 ± 0.96	6.15 ± 1.74
Second Wingate Test	8.96 ± 0.91	8.27 ± 1.62
Third Wingate Test	10.40 ± 0.91	9.71 ± 1.51

In the observer group, blood glucose increased from 5.32 ± 0.59 mmol/L to 6.28 ± 0.85 mmol/L after HIIE, and in the control group, blood glucose increased from 5.27 ± 0.61 mmol/L to 6.10 ± 0.72 mmol/L after HIIE. The main effect of group on blood glucose was not significant (*F* = 0.462, *P* = 0.500, *η²* = 0.010), indicating no statistically significant difference in the average blood glucose levels between the two groups across the entire pre- and post-test period. The main effect of “Times” was significant (*F* = 129.664, *P* < 0.001, *η²* = 0.730), meaning that post-test blood glucose levels were significant higher than pre-test levels. The interaction effect between “Times” and “Group” was not significant (*F* = 0.672, *P* = 0.416, *η²* = 0.014), indicating no statistically significant difference in the change in blood glucose between the two groups from pre- to post-test, and the trend of blood glucose increase was consistent. Bayesian RM-ANOVA results showed that after including the predictors, SMI (*β* = 0.25, 95% CI = 0.010.48) had a positive impact on blood glucose. The remaining predictors—group (*β* = 0.40, 95% CI=-0.971.82), FT3 (*β* = 0.01, 95% CI=-0.020.05), FT4 (*β* = 0.001, 95% CI=-0.010.01), and TSH (*β* = -0.16, 95% CI=-0.87~0.53)—had no statistically significant impact on blood glucose. (See **Figure 2**).

In the observer group, leptin increased from 3.40 ± 1.11 μg/L to 4.24 ± 1.27 μg/L after HIIE, and in the control group, leptin increased from 3.03 ± 1.01 μg/L to 4.30 ± 1.46 μg/L after HIIE. The main effect of group on leptin was not significant (*F* = 0.221, *P* = 0.640, *η²* = 0.005), indicating no statistically significant difference in the average leptin levels between the two groups across the entire pre- and post-test period. The main effect of “Times” was significant (*F* = 145.293, *P* < 0.001, *η²* = 0.752), meaning that post-test leptin levels were significant higher than pre-test levels. The interaction effect between “Times” and “Group” was significant (*F* = 6.181, *P* = 0.016, *η²* = 0.114), indicating that the trend of leptin increase was not consistent between the two groups from pre- to post-test, meaning there was a statistically significant difference in the change in leptin between the two groups. Bayesian RM-ANOVA results showed that after including the predictors, a significant difference existed between the two groups (*β* = -3.52, 95% CI=-5.72~-1.25), and TSH (*β* = 2.00, 95% CI = 0.863.11) had a positive impact on leptin. SMI (*β* = 0.03, 95% CI=-0.370.42), FT3 (*β* = -0.01, 95% CI=-0.060.05), and FT4 (*β* = 0.02, 95% CI=-0.000.03) had no statistically significant impact on leptin. (See **Figure 2**).

### 3.4 Changes in muscle-related indicators

**Figure 3:**
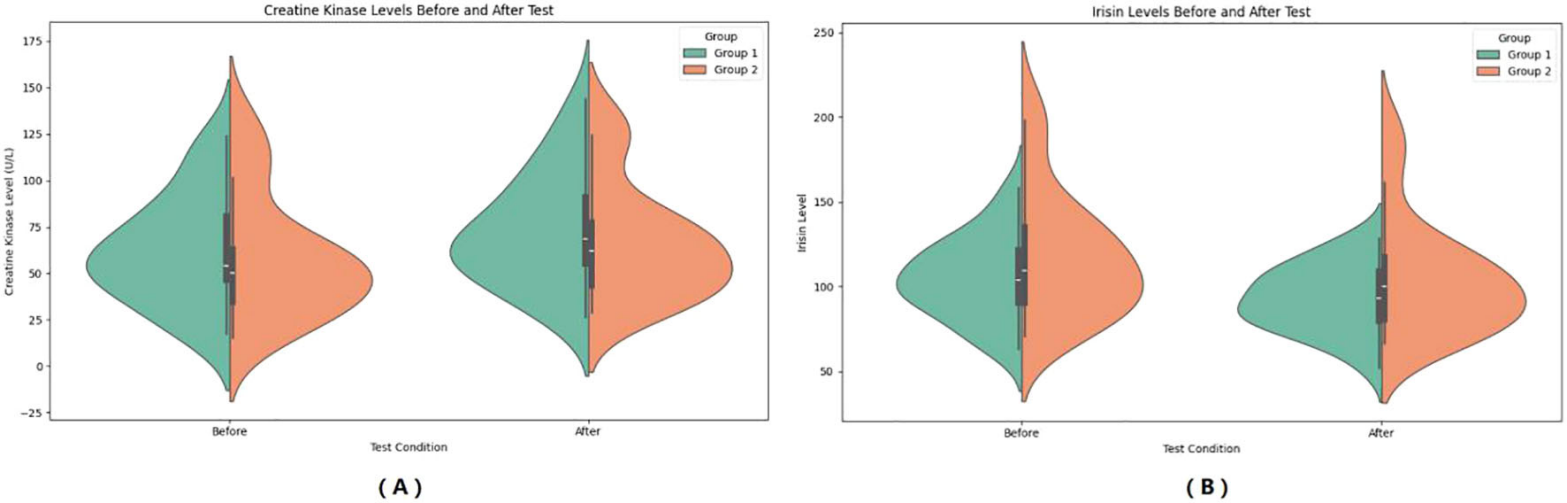
Pre- and Post-HIIE changes in muscle-related indicators: CK **(A)** and Irisin **(B)**. **(A)** Serum creatine kinase **(CK)** levels before and after the test. **(B)** Serum irisin levels before and after the test. Group 1: Control group; Group 2: Graves’ disease group. Data are expressed as violin plots showing the distribution and central tendency of each variable.

In the observer group, CK increased from 62.32 ± 28.88 IU/L to 73.27 ± 30.38 IU/L after HIIE, and in the control group, CK increased from 58.87 ± 32.73 IU/L to 66.82 ± 30.59 IU/L after HIIE. The main effect of group on CK was not significant (*F* = 0.348, *P* = 0.558, η²=0.007), indicating no statistically significant difference between the two groups in the average CK levels across the entire testing period. The main effect of “Times” was significant (*F* = 18.729, *P* < 0.001, *η²* = 0.281), showing that CK levels after the test were significant higher than before the test. The interaction effect between “Times” and group was not significant (*F* = 0.470, *P* = 0.496, *η²* = 0.010), suggesting no statistically significant difference in the change in CK levels between the two groups from pre- to post-test, and that the upward trend in CK was consistent. Bayesian RM-ANOVA results showed that after including the covariates, none of the predictors—group (*β* = -2.03, 95%CI=-11.307.12), SMI (*β* = 0.58, 95%CI=-6.557.69), FT3 (*β* = 0.19, 95%CI=-1.121.50), FT4 (*β* = -0.21, 95%CI=-0.670.26), and TSH (*β* = -2.93, 95%CI=-10.06~4.31)—had a statistically significant effect on CK. (See **Figure 3**).

In the observer group, CK increased from 92.77 ± 19.49 ng/L to 73.27 ± 30.38 ng/L after HIIE, and in the control group, CK increased from 92.77 ± 19.49 ng/L to 104.62 ± 33.54 ng/L after HIIE. The main effect of group on irisin was not significant (*F* = 2.292, *P* = 0.137, *η²* = 0.046), indicating no statistically significant difference between the two groups in the average irisin levels across the entire testing period. The main effect of “Times” was significant (*F* = 57.247, *P* < 0.001, *η²* = 0.544), showing that irisin levels after the test were significant higher than before the test. The interaction effect between “Times” and group was not significant (*F* = 0.067, *P* = 0.797, *η²* = 0.001), suggesting no statistically significant difference in the change in irisin levels between the two groups from pre- to post-test, and that the upward trend in irisin was consistent. Bayesian RM-ANOVA results showed that after including the covariates, none of the predictors—group (*β* = 2.04, 95%CI=-7.3711.34), SMI (*β* = -3.25, 95%CI=-10.163.66), FT3 (*β* = -0.16, 95%CI=-1.411.08), FT4 (*β* = 0.06, 95%CI=-0.370.52), and TSH (*β* = 4.72, 95%CI=-2.42~11.88)—had a statistically significant effect on irisin. (See **Figure 3**).

### 3.5 Changes in inflammatory indicators

**Figure 4:**
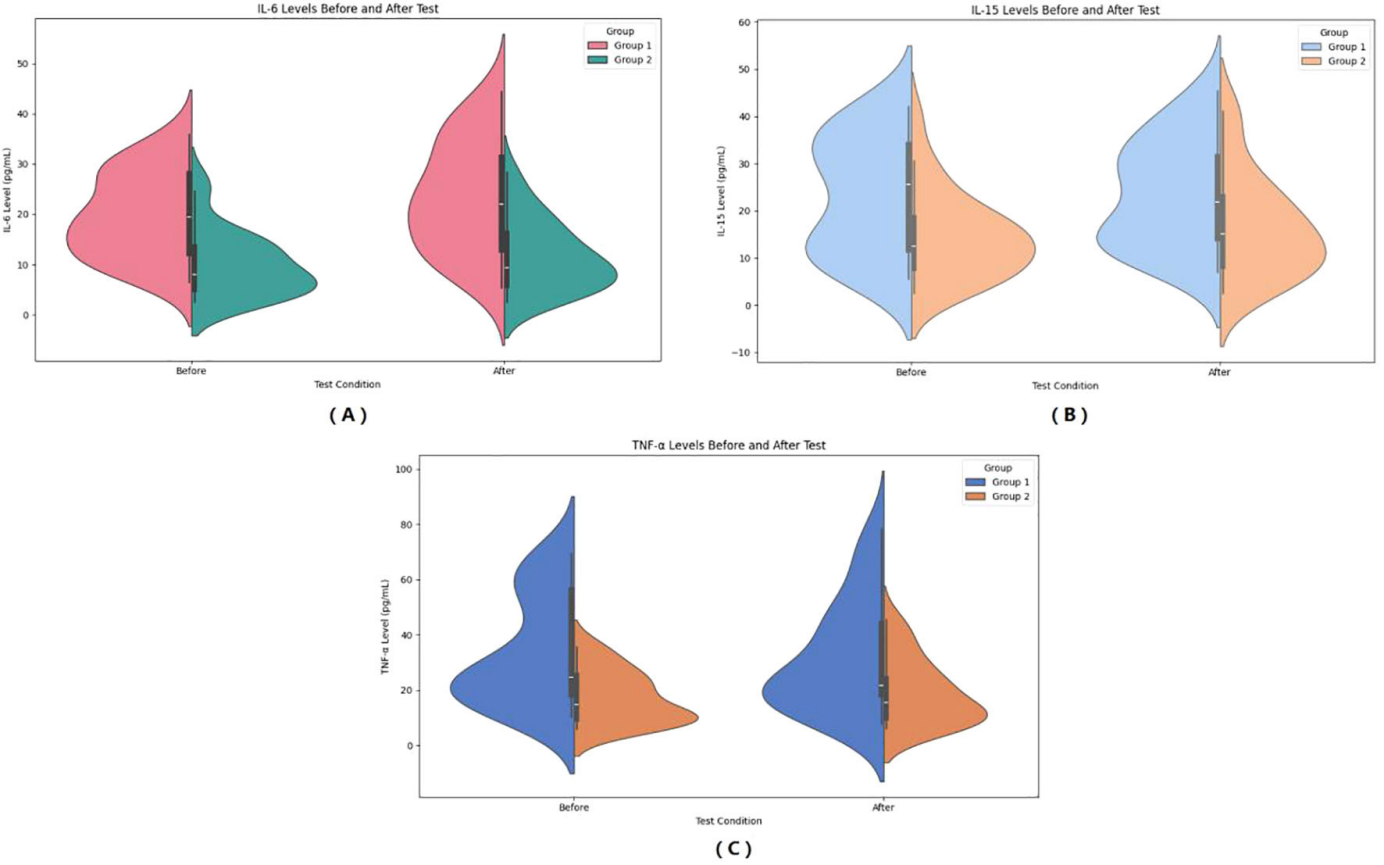
Pre- and post-HIIE changes in inflammatory indicators: IL-6 **(A)**, IL-15 **(B)**, and TNF-α **(C)**. **(A)** Serum IL-6 levels before and after the test. **(B)** Serum IL-15 levels before and after the test. **(C)** Serum TNF-α levels before and after the test. Group 1: Control group; Group 2: Graves’ disease group. Data are shown as violin plots, demonstrating data distribution, median, and variability before and after exercise.

In the observer group, IL-6 increased from 19.74 ± 8.43 mmol/L to 23.45 ± 10.94 mmol/L after HIIE, and in the control group, IL-6 increased from 9.96 ± 6.43 mmol/L to 11.65 ± 6.90 mmol/L after HIIE. The main effect of group on IL-6 was significant (*F* = 21.435, *P* < 0.001, *η²* = 0.309), indicating a statistically significant difference between the two groups in the average IL-6 levels across the entire testing period. The main effect of “Times” was significant (*F* = 44.448, *P* < 00.001, *η²* = 0.481), showing that IL-6 levels after the test were significant higher than before the test. The interaction effect between “Times” and group was significant (*F* = 6.155, *P* = 0.017, *η²* = 0.114), suggesting a statistically significant difference in the change in IL-6 levels between the two groups from pre- to post-test, and that the upward trend in IL-6 was inconsistent. Bayesian RM-ANOVA results showed that after including the covariates, none of the predictors—group (*β* = -1.08, 95%CI=-8.806.73), SMI (*β* = -1.38, 95%CI=-4.121.42), FT3 (*β* = 0.12, 95%CI=-0.270.49), FT4 (*β* = 0.05, 95%CI=-0.080.18), and TSH (*β* = -2.49, 95%CI=-6.77~1.80) had a statistically significant effect on IL-6. (See **Figure 4**).

In the observer group, IL-15 increased from 23.21 ± 12.31 mmol/L to 23.53 ± 11.23 mmol/L after HIIE, and in the control group, IL-15 increased from 14.62 ± 9.08 mmol/L to 16.55 ± 10.74 mmol/L after HIIE. The main effect of group on IL-15 was significant (*F* = 6.600, *P* = 0.013, *η²* = 0.121), indicating a statistically significant difference between the two groups in the average IL-15 levels across the entire testing period. The main effect of “Times” was not significant (*F* = 3.887, *P* = 0.054, *η²* = 0.075), showing no significant change in IL-15 levels after the test. The interaction effect between “Times” and group was not significant (*F* = 1.982, *P* = 0.166, *η²* = 0.040), suggesting no statistically significant difference in the change in IL-15 levels between the two groups from pre- to post-test, and that the trend of IL-15 change was consistent. Bayesian RM-ANOVA results showed that after including the covariates, none of the predictors—group (*β* = -2.43, 95%CI=-10.875.78), SMI (*β* = -0.06, 95%CI=-3.553.47), FT3 (*β* = -0.07, 95%CI=-0.570.42), FT4 (*β* = 0.10, 95%CI=-0.070.27), and TSH (*β* = -0.87, 95%CI=-5.64~3.95) had a statistically significant effect on IL-15. (See **Figure 4**).

In the observer group, TNF-α decreased from 33.79 ± 19.67 mmol/L to 31.83 ± 19.96 mmol/L after HIIE, and in the control group, TNF-α increased from 17.22 ± 9.34 mmol/L to 19.10 ± 11.77 mmol/L after HIIE. The main effect of group on TNF-α was significant (*F* = 11.808, *P* = 0.001, *η²* = 0.197), indicating a statistically significant difference between the two groups in the average TNF-α levels across the entire testing period. The main effect of “Times” was not significant (*F* = 0.001, *P* = 0.977, *η²* < 0.001), showing no significant change in TNF-α levels after the test. The interaction effect between “Times” and group was not significant (*F* = 1.817, *P* = 0.184, *η²* = 0.036), suggesting no statistically significant difference in the change in TNF-α levels between the two groups from pre- to post-test. Bayesian RM-ANOVA results showed that after including the covariates, none of the predictors—group (*β* = -2.28, 95%CI=-11.126.72), SMI (*β* = -2.81, 95%CI=-7.562.14), FT3 (*β* = 0.30, 95%CI=-0.421.04), FT4 (*β* = -0.04, 95%CI=-0.290.21), and TSH (*β* = -3.28, 95%CI=-8.94~2.25) had a statistically significant effect on TNF-α. (See **Figure 4**).

### 3.6 Bayesian network analysis results

**Figure 5:**
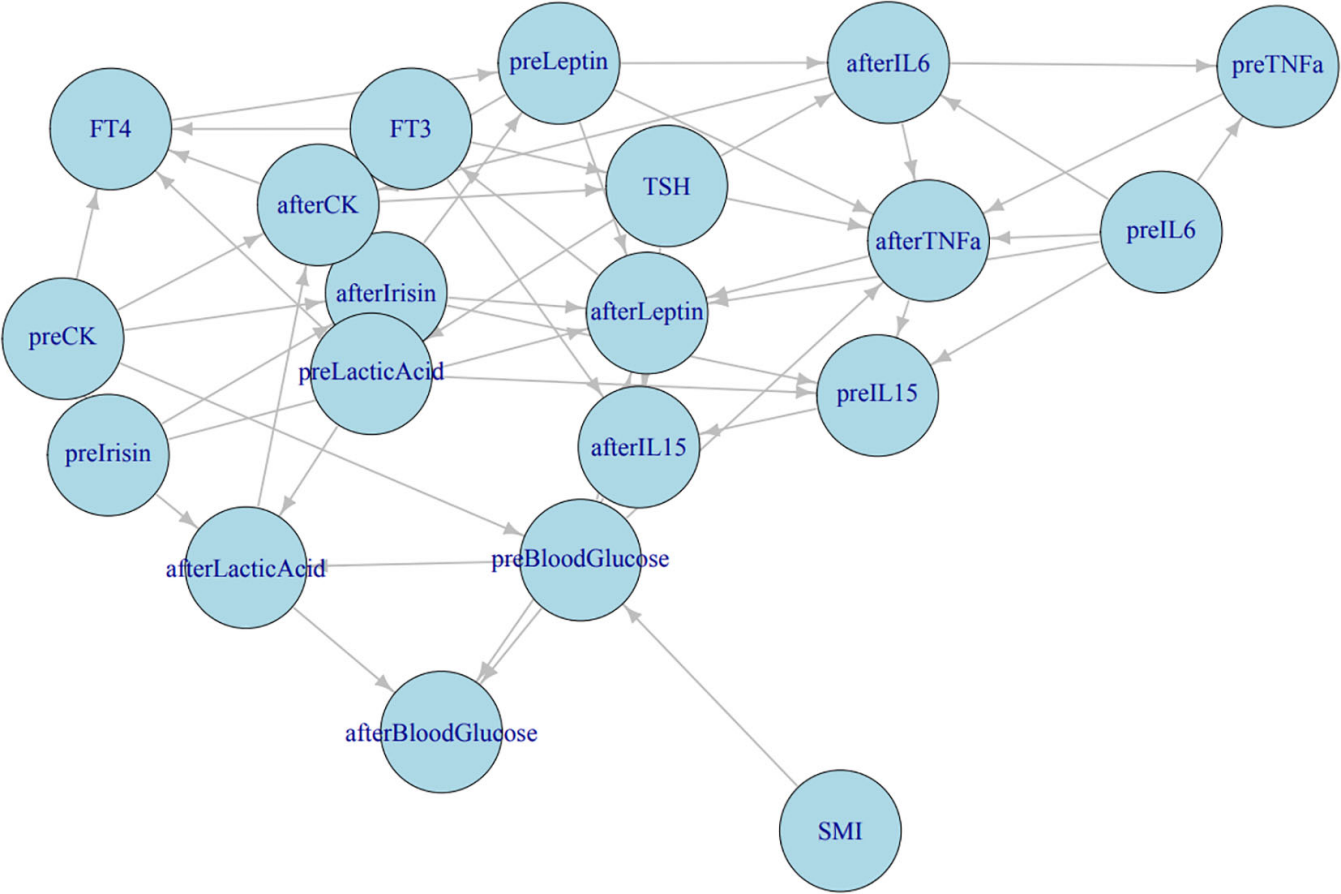
Bayesian network analysis results. Correlation network among thyroid function, metabolic, and inflammatory indicators. The directed acyclic graph illustrates the interrelationships between serum cytokines (IL−6, IL−15, TNF−α), myokines (irisin, leptin), thyroid hormones (FT3, FT4, TSH), and metabolic indicators (blood glucose, lactic acid, CK, SMI). “pre−” represents measurements before HIIE, and “after−” denotes measurements after HIIE. Arrow directions indicate the direction of correlation or influence derived from Bayesian network analysis.

We constructed comprehensive Bayesian networks for both the observation group and the control group. Each network comprised 23 nodes and several edges, with nodes representing different variables, including SMI, FT3, FT4, TSH, and various parameters observed during HIIE. The results indicate that FT3 may influence post-HIIE IL-15; FT4 affects post-HIIE IL-6 release by influencing pre-HIIE leptin, and the increase in post-HIIE IL-6 may, in turn, affect TNF-α. TSH’s influence on post-HIIE IL-6, IL-15, and TNF-α suggests its significant role in regulating inflammatory responses. SMI may affect pre-exercise blood glucose levels and indirectly influence post-exercise blood glucose levels. After exercise, the body’s ability to process blood glucose might be influenced by SMI. A higher SMI may contribute to more effective regulation of blood glucose levels post-exercise. (See **Figure 5**).

Structural learning of the Bayesian network models revealed complex relationships among FT3, FT4, and TSH within the observation group’s network, with significant interrelationships with multiple other biomarkers. These variables play a crucial role in regulating thyroid function and related physiological states. In contrast, the control group’s network structure was simpler and more stable, with FT3, FT4, and TSH being relatively stable and having smaller node degrees, indicating a weaker influence on the overall network. This reflects the characteristics of hormonal balance in healthy individuals. (See Graphical Abstract).

## 4 Discussion

This study aimed to systematically evaluate the similarities and differences in immunometabolic factors between female Graves’ hyperthyroidism patients and healthy controls after HIIE. The findings reveal distinctions and commonalities between hyperthyroid patients and healthy individuals in acute exercise performance and acute exercise-induced immunometabolic responses, further exploring potential physiological associations.

Baseline data showed that, besides significant differences in FT3, FT4, and TSH levels, the observation group also exhibited a significant lower SMI compared to the control group. This aligns with previous research indicating that emaciation in hyperthyroid patients primarily involves muscle loss, meaning hyperthyroidism is often accompanied by muscle wasting. This could be attributed to an imbalance in the regulation of protein synthesis and catabolism by thyroid hormones (18, 19). Thyroid hormones promote protein degradation and inhibit protein synthesis through various mechanisms, leading to accelerated muscle protein breakdown, consequently causing muscle atrophy and decreased strength (19, 20). This difference in muscle mass might potentially affect exercise performance even at the baseline stage, although this study did not observe significant differences in exercise power. This might be due to the recruitment of newly diagnosed Graves’ hyperthyroidism patients, where muscle loss could be an early stage whose functional impact has not yet fully manifested. As the disease progresses, if hyperthyroidism is not effectively controlled, muscle loss may further worsen, at which point a decline in exercise power could become more pronounced. Apart from the differences in thyroid hormones and SMI, no significant differences were found between the two groups in conventional metabolic and cardiovascular indicators such as age, BMI, blood glucose, cholesterol, triglycerides, low-density lipoprotein, high-density lipoprotein, and blood pressure. This ensures that the observed differences in the study results are more likely attributable to the hyperthyroid state itself and its associated muscle changes, rather than the influence of other confounding factors, thereby enhancing the reliability of the study conclusions.

### 4.1 Comparison of HIIE exercise power and attenuation pattern

In terms of HIIE exercise power, this study found no significant differences in the absolute levels of either peak power or mean power between the two groups across three Wingate tests. However, for mean power, we observed a significant “Times by group interaction,” indicating a notable difference in the trend of mean power decline between the two groups. Specifically, as the number of tests increased, both peak power and mean power in both groups significant decreased, which is consistent with the characteristics of HIIE, where accumulated exercise fatigue leads to a decline in exercise performance.

It is worth noting that despite the observation group having a significant lower SMI than the control group, their absolute exercise power was not significant lower than that of healthy controls. This seems to somewhat deviate from our initial hypothesis (that muscle loss in hyperthyroidism might lead to decreased exercise endurance). Besides considering that the impact of muscle loss in newly diagnosed patients may not yet be fully manifested, one possible explanation is that, although hyperthyroid patients experience muscle loss, their elevated basal metabolic rate might, to some extent, compensate for the insufficient muscle mass, allowing them to maintain similar power output to healthy individuals during short, high-intensity explosive exercise. The excessive secretion of thyroid hormones associated with hyperthyroidism can enhance mitochondrial oxidative phosphorylation, thereby increasing basal metabolism and energy production (21), which may provide additional energy support for short-duration high-intensity exercise.

Furthermore, the Wingate test primarily reflects anaerobic exercise capacity. Although the initial explosive power of hyperthyroid patients was similar in this test, the differing trend in mean power decline might suggest that hyperthyroid patients exhibit different patterns of fatigue accumulation or recovery ability during repeated bouts of high-intensity exercise. This aligns with observations in a study by Kimura et al. (22), which found significant effects of hyperthyroidism on exercise efficiency and cardiopulmonary function, indicating that during sustained high-intensity exercise, hyperthyroid patients may need to consume more oxygen to produce the same power, or output less power at the same oxygen consumption, leading to faster fatigue accumulation. Studies by Kahaly et al. (23) and Irace et al. (24) further support this view. Kahaly et al. (23) observed that during exercise, hyperthyroid patients had significant smaller increases in minute ventilation, oxygen uptake, oxygen pulse, and heart rate compared to their euthyroid state, and both anaerobic threshold and work performed at maximal exercise were reduced. Irace et al. (24) also found that hyperthyroid patients had shorter exercise durations, lower work rates and oxygen consumption at the anaerobic threshold, and significant reduced oxygen pulse. All these findings reflect the negative impact of hyperthyroidism on the cardiovascular system and oxygen delivery capacity. Even at intensities below or near the anaerobic threshold, patients’ cardiopulmonary function is limited, making it difficult to sustain prolonged or high-intensity exercise. This is consistent with the trend of decreasing mean power observed in the Wingate test, suggesting impaired adaptability of the circulatory and respiratory systems in hyperthyroid patients when coping with sustained high-intensity exercise.

Bayesian repeated measures ANOVA further confirmed that the included predictor variables, such as thyroid hormones and SMI, had no statistically significant impact on either peak power or mean power. This suggests that in short-duration explosive exercises like the Wingate test, thyroid status and SMI may not be the primary factors determining “initial” exercise power. However, this does not mean that these parameters have no effect on “sustained” anaerobic exercise capacity. While hyperthyroidism may not directly affect the “peak” output of short-duration explosive power, its long-term impact on exercise efficiency, cardiopulmonary function, and oxygen delivery capacity will indirectly lead to a decrease in “mean power” and accelerated fatigue accumulation during high-intensity repeated exercise.

### 4.2 Changes in HIIE-induced metabolic indicators and their implications

The results of this study showed no significant differences between the two groups of participants in the absolute levels of blood lactate and blood glucose, nor in their post-HIIE elevation trends. The significant increase in blood lactate levels after exercise is a typical physiological response to high-intensity anaerobic exercise, aimed at inducing adaptation in skeletal muscle during strenuous activity, reflecting active glycolysis and lactate accumulation in muscles (25). Strenuous exercise also causes an increase in blood glucose, with the core mechanism being the substantial stimulation of hepatic glucose production by catecholamines and the relative restriction of muscle glucose utilization. Concurrently, the suppression of insulin secretion is offset by hyperglycemia and a decrease in insulin clearance, while during the recovery phase, catecholamine levels decline, leading to a restoration of insulin responsiveness and promoting blood glucose normalization (26). Both groups of participants exhibited similar patterns of change in blood lactate and blood glucose, indicating that under acute HIIE stress, glucose metabolism and anaerobic glycolysis responses in newly diagnosed hyperthyroid patients do not significant differ from healthy controls. This suggests that, in this study, for newly diagnosed patients, although hyperthyroidism affects the basal metabolic rate, the activation level of their glucose and lactate metabolic pathways in terms of substrate utilization induced by acute high-intensity exercise is similar to that of healthy individuals. Bayesian repeated measures ANOVA also supported this point; apart from SMI having a positive effect on blood glucose, other thyroid-related variables did not significant affect lactate and blood glucose. However, the results also indicated that individuals with higher SMI might have higher post-exercise blood glucose levels, which could be related to greater muscle mass consuming more glucose during exercise. Earlier studies also indicated that skeletal muscle increases glucose uptake to maintain muscle contraction during exercise (27).

Regarding leptin, both groups showed significant elevated leptin levels after HIIE, and Bayesian repeated measures ANOVA revealed a significant difference between the two groups, with TSH having a positive effect on leptin. Leptin is a hormone primarily secreted by adipose cells, involved in regulating energy balance and fat metabolism (28). Current research on the effect of exercise on leptin production still shows variability. Ishigaki et al. (29) and Zafeiridis et al. (30) reported no changes in serum leptin immediately after high-intensity endurance training and resistance exercise. In contrast, Pop et al. (31) showed a significant decrease in leptin levels in overweight women after exercise. Fisher et al. (32) found that serum leptin increased at the start of high-intensity exercise and decreased at the end of exercise, returning to control levels 139 minutes post-exercise. This study suggested that exercise-induced cortisol elevation might prevent the decrease in leptin and that factors influencing post-exercise leptin are complex, potentially affected by the subject’s health status and multiple other factors; thus, more research is needed to elucidate the mechanism.

Regarding differences in leptin levels under different thyroid states, existing research presents complex results. On one hand, several studies indicate that thyroid function itself does not seem to directly affect leptin secretion. For example, studies by Yaturu et al. (33) and Wahrenberg et al. (34) found that changes in thyroid function or the hyperthyroid state itself did not lead to abnormal leptin levels, suggesting that in some cases, the direct influence of thyroid hormones on leptin may not be significant. However, other studies provide different perspectives. Observations by Zimmermann-Belsing et al. (35) showed that leptin levels actually increased in treated hyperthyroid patients. This might suggest that certain factors during treatment or physiological changes during hyperthyroid recovery might influence leptin. Furthermore, the Bayesian analysis results suggest that the hyperthyroid state might affect exercise-induced leptin responses by influencing the relationship between TSH and leptin. This indicates that hyperthyroidism may alter the regulatory mechanisms of leptin in energy metabolism and exercise adaptation. A study by Yu et al. (36) on obese patients with type 2 diabetes mellitus and normal thyroid function revealed a significant positive correlation between serum levels of and TSH, and pointed out that leptin is an independent factor influencing TSH. Mechanistically, leptin secretion is modulated not only by adipose tissue mass but also by neuroendocrine signals—such as TRH and sympathetic nervous system activity—which may be disrupted or exaggerated in hyperthyroid states, potentially altering leptin’s feedback interactions with the hypothalamic–pituitary–thyroid axis (37). Overall, we speculate that elevated basal metabolic rate and muscle loss in hyperthyroid patients may further influence baseline leptin levels and its response during exercise. Therefore, the impact of thyroid functional status on leptin levels and regulation may not be solely direct, but rather involve a complex network of metabolism, inflammation, and TSH interactions, necessitating further in-depth research.

### 4.3 Dynamic changes in muscle-related indicators

CK and irisin are important indicators for assessing muscle damage and muscle metabolism. This study showed that both CK and irisin levels significant increase in both groups after HIIE, with no significant differences between the two groups or in their elevation trends. CK is an enzyme widely distributed in tissue cytoplasm and mitochondria, with the highest content in skeletal muscle. Strenuous exercise causes CK elevation, and the more intense the exercise, the higher the values. The appearance of CK in the blood is generally considered an indirect marker of muscle damage (38–40). Irisin is a newly discovered “exercise factor,” primarily secreted by skeletal muscle, involved in various physiological processes such as energy metabolism, fat browning, and improving insulin sensitivity. Exercise-induced irisin elevation can inhibit inflammation and is therefore considered one of the important molecular mechanisms for exercise’s health benefits (41, 42). Moreover, muscle changes caused by alterations in thyroid function can lead to changes in irisin levels, with hyperthyroid patients often having high levels of irisin, and elevated irisin levels possibly leading to altered energy metabolism in hyperthyroidism patients (41, 43). Previous studies have shown that irisin levels significant increase after acute exercise, including HIIE (44, 45), and our study consistently found the same.

Despite muscle loss in hyperthyroid patients, their CK and irisin responses after HIIE were similar to those of healthy controls. Although hyperthyroidism is known to cause muscle loss, our results suggest that even with potentially reduced muscle mass, the acute response patterns of muscle damage and myokine (such as irisin) secretion in hyperthyroid patients’ muscle tissue undergoing high-intensity stress may not significant differ from those in healthy individuals. This might mean that hyperthyroidism has a minor impact on the acute regulatory mechanisms of muscle damage repair and myokine secretion, or that the HIIE stimulus intensity was sufficient to elicit similar acute muscle damage and myokine secretion responses in both groups. Furthermore, the Bayesian analysis results failed to find significant effects of thyroid hormones and SMI on CK and irisin, further supporting this view. This suggests that under the acute stress of high-intensity exercise, the release of biochemical markers of muscle damage (CK) and myokines (irisin) may be determined more by the intensity and nature of the exercise itself, rather than being significant constrained by thyroid functional status or the extent of prior muscle loss. This has important implications for understanding muscle adaptability in hyperthyroid patients undergoing exercise interventions.

### 4.4 Response patterns of inflammatory indicators

This study primarily examined the changes in inflammatory cytokines IL-6, IL-15, and TNF-α before and after HIIE. Previous studies have demonstrated that these cytokines are closely associated with thyroid dysfunction and play critical roles in the pathogenesis and progression of Graves’ disease. Specifically, elevated levels of the pro-inflammatory cytokine IL-6 may promote B-cell differentiation and the production of thyroid-stimulating hormone receptor antibodies (TRAb), thereby exacerbating hyperthyroidism. IL-6 also contributes to immune imbalance by inducing Th17 differentiation and suppressing regulatory T cells (Treg), which disrupts immune tolerance and aggravates thyroid injury and hypermetabolic status. TNF-α, another potent pro-inflammatory mediator, amplifies the inflammatory response through the IL-17A pathway and cooperates with IL-1β to induce tissue damage. Moreover, the expression of IL-15 has been reported to be significantly associated with the pathogenesis and progression of thyroid-associated ophthalmopathy (46, 47). Because patients with hyperthyroidism frequently experience skeletal muscle loss, these cytokines may constitute key mechanistic links between endocrine dysfunction and muscle deterioration. IL-6 and TNF-α can promote muscle protein degradation, whereas IL-15 has been suggested to facilitate skeletal muscle protein synthesis (48, 49). Therefore, IL-6, IL-15, and TNF-α were selected as representative indicators in this study to explore the potential interplay among thyroid function, inflammatory state, and skeletal muscle metabolism.

The results showed a significant difference in the average level of IL-6 between the two groups, and IL-6 levels significant increase in both groups after HIIE. However, the trend of IL-6 elevation was inconsistent between the two groups, indicating a significant “Times by Group interaction.” While average levels of IL-15 and TNF-α also showed significant differences between the two groups, neither IL-15 nor TNF-α levels significant changed after HIIE, and their change trends were consistent between groups. Even a small total exercise volume can lead to an increase in IL-6 levels (1), and a study by Pérez-López et al. (50) also showed significant increases in plasma IL-6 and IL-15 after a high-intensity intermittent Wingate test. However, we did not find any research evaluating the acute pre- and post-exercise IL-6 response to HIIE in hyperthyroid patients. Research indicates that skeletal muscle can produce a large amount of IL-6 during exercise, and serum IL-6 levels significant increase after both aerobic and anaerobic exercise. Furthermore, IL-6 produced during exercise may promote fat breakdown for energy (51, 52). Additionally, IL-6 has both pro-inflammatory and anti-inflammatory activities mediated by different signaling pathways (2); anti-inflammatory IL-6 produced by resistance exercise can inhibit TNF-α (**5**, 53). Therefore, acute exercise-induced IL-6 elevation is considered a key link in its potential anti-inflammatory effects. In this study, the differing trend of IL-6 elevation between the two groups suggests that the hyperthyroid state might have affected the dynamic response of IL-6. Although Bayesian analysis failed to find a significant impact of thyroid hormones and SMI on IL-6, this might require a more complex or larger sample size for further exploration. Hyperthyroid patients have a high basal metabolic rate and may be in a state of chronic low-grade inflammation, and the markedly elevated baseline IL-6 levels in this group may act as a pre-activated inflammatory state, potentially modulating the magnitude and trajectory of the exercise-induced IL-6 surge, thereby shaping downstream metabolic and immune adaptations (54). Thus their inflammatory response patterns to acute exercise stress might differ from healthy individuals.

IL-15 is also a myokine, primarily involved in immune regulation and muscle growth. Hingorjo et al. (55) showed that after 3 minutes of stepping exercise, IL-15 levels were higher in lean individuals compared to overweight/obese individuals. Yargic et al. (56) found that acute exercise can also promote an increase in serum IL-15 levels. Tamura et al. (57) suggested that moderate to submaximal intensity exercise might be sufficient to significant increase circulating IL-15 levels, and vigorous high-intensity training is not necessarily required. This study also compared the change patterns of IL-15 and CK, finding that IL-15 quickly peaked at 10 minutes post-exercise and rapidly recovered, while CK peaked at 3 hours post-exercise. Therefore, the study concluded that the increase in IL-15 levels is due to active secretion caused by muscle contraction, rather than passive release due to exercise-induced muscle damage. In this study, we found that IL-15, while showing an increase after HIIE, did not reach statistical significance, contrasting with the acute elevation of IL-6. This is also consistent with Tamura et al.’s (57) finding that the peak elevation of IL-15 occurs 10 minutes post-exercise. This indicates specificity in the response patterns of different cytokines to acute HIIE.

A study by Zwetsloot et al. (6) showed that a single acute HIIT session can significant increase TNF-α expression. However, a rat study by Zuo et al. (58) indicated that acute exercise might not significant increase TNF-α levels, but TNF-α mRNA levels in soleus muscles significant increased. Thus, this study suggested that TNF-α might play a role in repair mechanisms such as inflammatory response, cell recruitment, and tissue remodeling after muscle damage, rather than just acute inflammation. In this study, TNF-α also showed no significant change after HIIE. This result further supports the view that acute exercise may not significant impact circulating TNF-α levels and prompts us to focus more on the dynamic changes of TNF-α in local muscle rather than systemic circulation.

In summary, HIIE primarily elicits acute release of IL-6, while the responses of IL-15 and TNF-α may require a longer time or different types of exercise stimuli. The observed differences in the average levels of IL-15 and TNF-α between the two groups might reflect differences in the basal inflammatory state of hyperthyroid patients.

### 4.5 Complex associations revealed by Bayesian network analysis

This study utilized Bayesian network analysis to learn network structures from data of both the observation group and the control group, revealing complex relationships between variables. Bayesian networks can capture conditional dependencies between variables, offering more comprehensive insights.

In the network of the observation group (hyperthyroid patients), FT3, FT4, and TSH exhibited complex interrelationships and significant interactions with multiple biomarkers. This underscores the central role of thyroid hormones in regulating the overall physiological state of hyperthyroid patients. Specifically, FT3 might influence post-HIIE IL-15 levels, suggesting that thyroid hormones may indirectly participate in muscle adaptation processes by affecting the immune system. FT4, by influencing pre-HIIE leptin, subsequently affects the release of post-HIIE IL-6, and the increase in post-HIIE IL-6 might, in turn, affect TNF-α. This forms a thyroid hormone-leptin-IL-6-TNF-α signaling axis, suggesting that thyroid function in hyperthyroid patients might, mediated by leptin, influence the release of exercise-induced inflammatory factors like IL-6 and TNF-α. The influence of TSH on post-HIIE IL-6, IL-15, and TNF-α further indicates its important role in regulating inflammatory responses. This differs from the traditional view that TSH primarily regulates thyroid hormone secretion and may suggest direct or indirect roles of TSH in immunometabolism. A study by Zhu et al. (59) also showed that in hyperthyroid patients, serum TNF-α was negatively correlated with TSH, and the two soluble TNF-α receptors, sTNF-R1 and sTNF-R2, were positively correlated with FT3 and FT4 levels, especially sTNF-R1, which was considered one of the important factors affecting FT3 levels. Reduced muscle cell responsiveness to insulin is a characteristic of insulin resistance (60). Muscle levels might influence pre-exercise blood glucose levels and indirectly affect post-exercise blood glucose levels, highlighting the importance of muscle mass in glucose homeostasis, especially under exercise stress, where higher SMI might contribute to more effective blood glucose regulation.

In contrast, the network structure of the control group (healthy individuals) was simpler and more stable, with FT3, FT4, and TSH being relatively stable and having smaller node degrees, indicating a weaker influence on the overall network. This reflects the characteristics of hormonal balance in healthy individuals, where thyroid function is in a steady state, and its direct influence on other metabolic and immune indicators is not as significant as the compensatory or pathological effects observed in hyperthyroid patients. This difference in network structural complexity between groups precisely reveals the remodeling and adaptive changes of the body’s internal regulatory network under the pathophysiological state of hyperthyroidism.

### 4.6 Summary

This study yielded meaningful insights but has several limitations. The sample size was relatively small (25 women per group) and restricted to newly diagnosed female Graves’ hyperthyroidism patients, which enhanced cohort homogeneity but inevitably reduced generalizability. The study adopted the Wingate test as the sole high-intensity intermittent exercise modality, focused solely on acute responses before and after HIIE without long-term intervention evaluation, did not assess certain key biomarkers and baseline variables, and excluded patients with prolonged disease duration or more severe conditions. These limitations have been addressed in later phases of our project through more diverse participant cohorts, expanded biomarker profiling, and extended follow-up measurements; however, due to differences in participants, these data are not presented here. The primary objective of this work was to perform a Bayesian re-analysis of the pilot-phase dataset to evaluate the feasibility and advantages of this statistical approach in this field. Notably, Bayesian statistics offer inherent advantages in small-sample studies, enabling more stable and information-rich inferences under limited data conditions. All subsequent datasets from our project have undergone Bayesian re-analysis and are being prepared for submission. We consider this study a “pathfinder” application of Bayesian methodology to the immunometabolic investigation of hyperthyroid sarcopenia, laying the methodological foundation for future large-scale, stratified analyses.

Despite the aforementioned limitations, this study is the first to systematically evaluate the dynamic changes in the immune-metabolic profile of hyperthyroid patients after sequential HIIE and compare them with a healthy control group. Particularly, through re-analysis using Bayesian statistics, it revealed complex network relationships between thyroid hormones, SMI, and exercise-induced immune-metabolic responses, providing new perspectives for a deeper understanding of exercise physiology and metabolic adaptation in hyperthyroid patients. These findings have important clinical guiding significance for directing hyperthyroid patients to undertake safe and effective exercise interventions, optimizing their exercise prescriptions, and improving prognosis.

## 5 Conclusion

This study is the first to systematically evaluate the immune-metabolic responses in female Graves’ hyperthyroid patients after HIIE. While muscle loss in newly diagnosed hyperthyroid patients did not significant affect short-term explosive power, it may weaken endurance for sustained high-intensity exercise; their glucose metabolism and anaerobic glycolysis responses were similar to those of healthy individuals. Crucially, the hyperthyroid state significant altered exercise-induced immune-metabolic dynamic responses, and thyroid hormones constructed a complex metabolic and immune regulatory network within hyperthyroid patients. These findings provide new perspectives for a deeper understanding of the exercise physiological characteristics of hyperthyroid patients, emphasizing the need to comprehensively consider the potential impact of hyperthyroidism on the body’s complex physiological network when developing personalized exercise prescriptions.

## Data Availability

The raw data supporting the conclusions of this article will be made available by the authors, without undue reservation.
